# Incidence and characteristics of stroke in Zanzibar–a hospital-based prospective study in a low-income island population

**DOI:** 10.3389/fneur.2022.931915

**Published:** 2022-07-28

**Authors:** Jutta M. Adelin Jørgensen, Dirk Lund Christensen, Karoline Kragelund Nielsen, Halima Saleh Sadiq, Muhammad Yusuf Khan, Ahmed M. Jusabani, Richard Walker

**Affiliations:** ^1^Department of Public Health, University of Copenhagen, Copenhagen, Denmark; ^2^Mnazi Mmoja Referral Hospital, Zanzibar, Tanzania; ^3^Health Promotion Research, Steno Diabetes Center Copenhagen, Herlev, Denmark; ^4^Faculty of Medical Sciences, Newcastle University, Newcastle upon Tyne, United Kingdom; ^5^Radiology Department, The Aga Khan Hospital, Dar es Salaam, Tanzania; ^6^Department of Medicine, North Tyneside General Hospital, Northumbria Healthcare NHS Foundation Trust, Newcastle upon Tyne, United Kingdom; ^7^Population Health Sciences Institute, Newcastle University, Newcastle upon Tyne, United Kingdom

**Keywords:** acute stroke, sub-Saharan Africa, hypertension, CVD risk factors, low-income countries (LICs), incidence rate, Zanzibar

## Abstract

**Background:**

Stroke in adults is a critical clinical condition and a leading cause of death and disability globally. Epidemiological data on stroke in sub-Saharan Africa are limited. This study describes incidence rates, stroke types and antecedent factors among patients hospitalized with stroke in Zanzibar.

**Methods:**

This was a prospective, observational study of stroke patients at hospitals in Unguja, Zanzibar. Socioeconomic and demographic data were recorded alongside relevant past medical history, medicine use and risk factors. The modified National Institute of Health Stroke Scale (mNIHSS) was used to assess admission stroke severity and, when possible, stroke was confirmed by neuroimaging.

**Results:**

A total of 869 stroke admissions were observed from 1^st^ October 2019 through 30^th^ September 2020. Age-standardized to the World Health Organization global population, the yearly incidence was 286.8 per 100,000 adult population (95%CI: 272.4–301.9). Among these patients, 720 (82.9%) gave consent to participate in the study. Median age of participants was 62 years (53–70), 377 (52.2%) were women, and 463 (64.3%) had a first-ever stroke. Known stroke risk factors included hypertension in 503 (72.3%) patients, of whom 279 (55.5%) reported regularly using antihypertensive medication, of whom 161 (57.7%) had used this medication within the last week before stroke onset. A total of 460 (63.9%) participants had neuroimaging performed; among these there was evidence of intracerebral hemorrhage (ICH) in 140 (30.4%). Median stroke severity score using mNIHSS was 19 (10–27).

**Conclusion:**

Zanzibar has high incidence of hospitalization for stroke, indicating a very high population incidence of stroke. The proportion of strokes due to ICH is substantially higher than in high-income countries. Most stroke patients had been in contact with health care providers prior to stroke onset and been diagnosed with hypertension. However, few were using antihypertensive medication at the time of stroke onset.

www.ClinicalTrial.gov registration NCT04095806.

## Introduction

Stroke is a major cause of disability and the second leading cause of death worldwide (1, 2). Worldwide, one in four adults will experience a stroke during their lifetime (3). Over the past two decades, increasing knowledge and improved treatment of stroke have led to increased survival and reduced dependency among stroke patients in high-income countries (HICs) (4). Prevalence of uncontrolled hypertension–the most significant modifiable risk factor for stroke–has been reduced in many of these countries (5), and a decrease in stroke incidence has been observed globally (3).

At the same time, in low- and middle-income countries (LMICs), the burden of stroke is increasing (6, 7) with >75% of global stroke-related deaths and >80% of disability-adjusted life-years occurring in LMICs (3). Premature mortality and disability resulting from chronic disease have considerable economic and social implications, due to the impact on working populations, family units, and surrounding communities (8).

In sub-Saharan Africa (SSA), the prevalence rate of high blood pressure (BP) is among the highest in the world (5, 9). In the last decades, stroke incidence rates in SSA seem to be increasing (9, 10), and stroke is now recognized as an important cause of death also in SSA (10). One of the few existing population-based stroke incidence studies from SSA was conducted in Tanzania and found an age-standardized incidence rate up to 316 per 100,000 population (11).

The epidemiology of stroke in SSA has not been characterized optimally, and there are many questions still unanswered, including incidence, subtypes of stroke, risk factors and the strength of their association with stroke incidence and mortality. The Zanzibar Stroke Study is a prospective study investigating hospital-based incidence and stroke characteristics and outcomes in a stable population in a well-defined geographical area with very limited ability to seek care elsewhere during medical emergencies. The aim of the present study is to assess the hospital-based incidence rate of stroke, stroke types, severity, and known risk factors.

## Materials and methods

### Study design, setting and participants

The study was an observational prospective cohort study and as a result no additional care, finance or travel arrangements were offered to the participants. All individuals ≥18 years with permanent residence in Unguja, Zanzibar, who presented within 30 days of first-ever, or recurrent, stroke and were admitted to hospital or developed stroke whilst admitted to hospital with another diagnosis, were eligible for participation. Recruitment occurred uninterrupted from 1^st^ October 2019 through 30^th^ September 2020.

Zanzibar, a semi-autonomous part of the United Republic of Tanzania with its own administration including Ministry of Health, consists of the two islands Unguja and Pemba, with surrounding smaller islets. In 2019, the population was 1,625,589 individuals of whom 66.5% were living on the island of Unguja, and 52.6% of the population were below 20 years of age (12). Health services are provided free of charge at point-of-care in the public health facilities, and the entire population lives within 5 km of a public health facility (13).

Unguja has three public hospitals, which provide services free of charge. One of these, Mnazi Mmoja Hospital (MMH), has computed tomography (CT) imaging and magnetic resonance imaging (MRI) facilities. There are three private hospitals, one of which has a CT imaging facility. The research team was not granted access to the two military hospitals on the island. These two hospitals are smaller and with low in-patient volume, and stroke patients from the military hospitals were normally, but not always, referred to one of the two hospitals with neuroimaging capacity, and hence enrolled in the study upon arrival there.

### Case definition

Diagnosis of stroke was based on World Health Organization (WHO) clinical definition: “A focal (or at times global) neurological impairment of sudden onset, and lasting more than 24 h (or leading to death), and of presumed vascular origin” (14). Participants who responded to having had no prior episode of stroke were classified to have a first-ever stroke. A clinical stroke diagnosis was, whenever possible, confirmed by neuroimaging. The study aimed to register all incident strokes during the study period including first-ever as well as recurrent strokes. Subjects were excluded from the study if the neuroimaging led to a diagnosis other than stroke. The definitions used to describe the manifestations of stroke on non-contrast CT scans were acute (0–24 h), subacute (1–14 days), and chronic (>14 days). If no abnormalities were detected on the scan, the underlying mechanism was suspected to be an ischemic insult, while for those with a mixed stroke subtype the underlying cause was assumed to be an ischemic event with secondary hemorrhagic transformation, and in chronic infarcts the underlying stroke mechanism could not be determined.

### Recruitment, data collection

The research team was based at MMH from where they conducted daily visits to the general medical wards, private wards, and the intensive care unit to review new admissions, alongside checking medical records, and register and treatment books to identify patients with stroke symptoms. Other wards were visited regularly, and staff contacted the research team if there was a suspected stroke case. The research team also contacted doctors at the other five study sites daily, to check for new stroke admissions in the previous 24 h.

Potential participants were approached within 24 h of hospital admission and assessed by one of the research team members. If unsure of any diagnosis, the case would be discussed, with the Principal Investigator (JMAJ) making the final decision. After written consent was obtained, interviews were conducted upon enrollment and on the day of discharge from hospital.

Data were collected and managed using an electronic data capture tool and database developed and hosted by Vervig®. All identified stroke patients were entered into the stroke register with age, gender, and a unique study number.

### Data variables

The interviewer-administered structured questionnaire was built on the WHO STEPwise approach to stroke surveillance (14), and modified based on the Stroke Investigative Research and Education Network (SIREN) Phenomics protocol (15, 16), and general recommendations from the International Stroke Genetics Consortium (17), and World Stroke Organization (18) (Supplementary Material 1).

Individual level data on demographic and socioeconomic variables were accrued during enrollment by interview of patient or next of kin, as well as information on pre-stroke medical history and physical disability using the modified Rankin scale (mRS), and self-reported behavioral risk factors. Information on any previous stroke was obtained, with only those without a prior stroke being classified as having a first-ever stroke.

Tobacco smoking was categorized as current smoker, ex-smoker, or never smoked tobacco. Alcohol consumption was categorized as currently drinking daily, currently drinking alcohol but not daily, or not (within the past 12 months) drinking alcohol. Daily average physical activity was categorized as at/above or below 30 min of moderate physical activity. Daily intake of fish, meat or leafy green vegetables was characterized as minimum one serving of fish daily or there was respective daily intake of meat or leafy green vegetables.

Sedentary lifestyle was defined as being sedentary on average 8 h or more a day, nighttime sleep excluded (19). Previous relevant illnesses, such as prior diagnosis of hypertension, diabetes, human immunodeficiency virus (HIV) or sickle cell disease, were recorded. Other information collected included regular use of medicine for hypertension and diabetes as well as use of this medication within the week prior to stroke onset, and stroke symptom presentation.

All participants had their waist circumference (WC) measured using a tape measure at midline level (WC-mid) between the inferior margin of the ribs and the superior border of iliac crest, with recording of patient's position during measurement and defined as increased if ≥80 cm for females and ≥94 cm for males (20, 21). For supine position, a conversion factor was used to provide a corrected standing WC-mid value (22). Stroke severity was assessed using the modified National Institute of Health Stroke Scale (mNIHSS) within 48 h of admission. Administers of the mNIHSS had undergone certification to use the scale. A score of 1–4 was defined as minor stroke, 5–15 moderate stroke, 15–20 moderately severe stroke, and 21–32 severe stroke.

Data for all relevant clinical, radiological, laboratory tests, and treatment given were extracted from the patient medical record (see Supplementary Material 2 for entire list of variables, definitions, source of data and details of assessment). For those participants who had neuroimaging performed (using either Siemens Somaton emotion (series 29271) VB42B or Siemens Somaton go.Top (series 119175) or Siemens 1.5T Magneton Aera), a digital copy of any neuroimaging performed was obtained.

## Statistical analysis

For the descriptive analysis, unweighted numbers and proportions are displayed. Categorical variables are expressed as percentage, and continuous variables by mean and standard deviation (SD) or median and interquartile range (IQR) in case of skewed data.

Missing data were interpreted as true missing if the questionnaire section was not completed (no data entered) and were hence excluded from analyses of the missing variable(s).

For incidence calculations, the crude number of admitted patients with a stroke diagnosis in each 10-year age-band was used with the denominator being the official population projection for 2019 (23). For calculation of incidence of first-ever stroke, we assumed the proportion of first vs. recurrent stroke was the same among participants as among non-participants. Standardization to the WHO world standard population was done by use of the direct method. We calculated 95% confidence intervals (CI) for crude rates, for age- and sex-specific rates, and for age-adjusted incidence rates. Patients with missing age (*n* = 56) or sex (*n* = 2) were distributed proportionally to the existing crude numbers in each age-band to enable age- and sex-specific calculations. Based on assumption of Poisson distribution 95% confidence intervals were calculated. All analyses were undertaken using STATA version 16.0 (StataCorp, USA).

## Results

### Incidence rate

**Figure 1:**
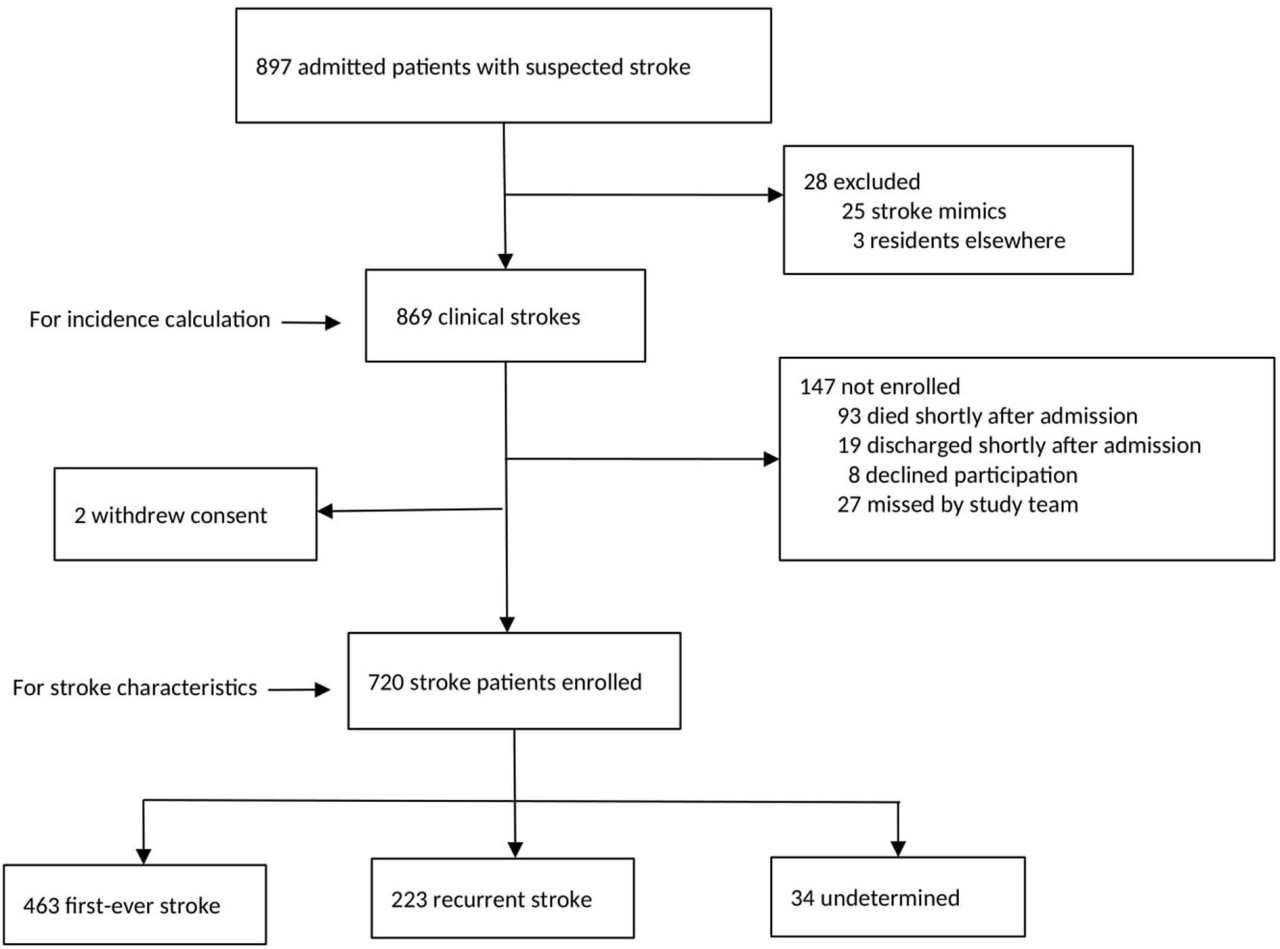
Analytic samples for incidence calculation and participant characteristics.

There was a total of 897 hospital admitted patients aged ≥20 years initially identified with a diagnosis of stroke according to the study criteria, of whom 25 were excluded as their final diagnosis was not stroke (based on neuroimaging), and 3 individuals were excluded as they were not residents in Unguja at the time, leaving a total of 869 individuals (see Figure 1).

The crude annual hospital-based incidence of any stroke (first-ever or recurrent) using Zanzibar population data was 169.6 (95% CI 158.5–181.2) per 100 000 adults, and incidence of first-ever stroke was 114.2 (95% CI 105.1–123.8) per 100 000 adults (Table 1). Women had slightly higher age-specific incidence rates than men in the younger age groups, only significant for first-ever stroke (*p* = 0.027). When standardized to the WHO world adult population, the incidence rate of any stroke was 286.8 (95% CI: 272.4–301.9) per 100,000 adults.

**Table 1:** Age- and sex-specific yearly incidence rates (IR) per 100.000 adults for **(A)** any stroke and **(B)** first-ever stroke standardized to the WHO world adult population.

	**Men**				**Women**			**Total**	
	***n***	**IR**	**(95% CI)**	***n***	**IR**	**(95% CI)**	***n***	**IR**	**(95% CI)**
**(A)** Age- and sex-specific yearly stroke incidence rates per 100.000 adults									
20–44 years	33	19.81	(13.5–27.5)	58	29.9	(22.6–38.4)	92	25.3	(20.3–30.9)
45–54 years	80	230.97	(182.8–287.3)	84	219.8	(174.7–271.5)	164	225.4	(192.1–262.6)
55–64 years	124	542.39	(450.2–645.8)	119	495.5	(409.5–591.9)	243	518.8	(455.3–588.0)
65–74 years	113	1,073.89	(881.1–1,286.5)	115	1,207.8	(997.0–1,448.3)	228	1,133.4	(990.2–1,289.7)
75+ years	62	1,367.32	(1,041.7–1,741.9)	81	1,618.1	(1,282.7–2,010.5)	143	1,500.9	(1,263.0–1,765.2)
All ages	412	171.19	(155.1–188.5)	457	168.1	(153.1–184.3)	869	169.6	(158.5–181.2)
Age-adjusted rate …			…			…	286.8	(272.4–301.9)
**(B)** Age- and sex-specific yearly first-ever stroke incidence rates per 100.000 adults									
20–44 years	21	12.2	(7.3–18.4)	46	23.6	(17.3–31.4)	66	18.3	(14.0–23.1)
45–54 years	60	174.5	(132.9–224.1)	52	137.7	(103.0–180.4)	112	154.9	(127.1–185.7)
55–64 years	74	325.3	(254.8–407.3)	76	315.0	(248.0–394.6)	149	319.6	(269.5–374.0)
65–74 years	84	802.7	(637.0–988.8)	86	896.0	(714.2–1,104.2)	170	846.0	(721.6–980.9)
75+ years	45	981.3	(710.0–1,307.1)	42	847.9	(607.8–1,140.0)	87	910.5	(725.5–1,118.7)
All ages	284	118.0	(104.7–132.6)	302	111.1	(98.6–124.0)	585	114.2	(105.1–123.8)
Age-adjusted rate …			…			…	192.2	(180.4–204.6)

### Study participants

Among the stroke patients, 720 (82.9%) consented to, and were enrolled in, the stroke study. All participants were ≥20 years. Majority of non-participants died shortly after admission and before the research team had identified and approached them (see Figure 1). Baseline features of the study cohort are shown in Tables 2, 3.

**Table 2:** Socio-demographic characteristics of study participant.

	**Any stroke (*****N*** = **720)**		**First-ever stroke (*****N*** = **463)**	
	***n*** **(or median)**	**% (or IQR)**	***n*** **(or median)**	**% (or IQR)**
**Demographic characteristics**				
**Age**	702		454	
Below 40 years	28	4.0%	23	5.1%
40 years and above	674	96.0%	431	94.9%
Median age (IQR)	62	53–70	62	52–70
**Gender**	720		463	
Female	377	52.2%	240	51.8%
Male	343	47.8%	223	48.2 %
**Education**	687		463	
< Primary school completed	200	27.8%	126	27.2%
Completed primary school	194	26.9%	132	28.5%
Completed secondary school	180	25.0%	123	26.6%
Level of education unknown	146	20.3%	82	17.7%
**Employment**	691		457	
Employed	70	10.1%	52	11.4%
Self-employed	201	29.1%	144	31.5%
Unemployed/unpaid/retired	420	60.8%	261	57.1%
**Covered by health insurance**	689		457	
No	590	85.6%	389	85.1%
Yes	99	14.4%	68	14.9%

**Table 3:** Pre-stroke condition and known risk factors.

	**Any stroke (*****N*** = **720)**		**First-ever stroke (*****N*** = **463)**	
	***n*** **(or median)**	**% (or IQR)**	***n*** **(or median)**	**% (or IQR)**
**Pre-admission status**				
**Previous stroke**	720			
Yes	223	31.0%		
No	463	64.3%		
Unsure/unknown	34	4.7%		
**Modified rankin scale**	720		463	
No disability	463	64.3%	349	75.4%
No significant disability	46	6.4%	31	6.7%
Slight disability	24	3.3%	10	2.2%
Moderate disability, walks unassisted	79	11.0%	29	6.3%
Moderate disability, walks assisted	47	6.5%	18	3.9%
Severe disability / bedridden	29	4.0%	8	1.7%
Unknown	32	4.4%	18	3.9%
**Hypertension (HTN)**	696		463	
Diagnosed with HTN	503	72.3%	308	66.5%
No prior diagnosis of HTN	193	27.7%	155	33.5%
Regularly uses antihypertensive medication^*^	503		308	
Yes	279	55.5%	172	55.8%
No	153	30.4%	98	31.8%
Unsure/unknown	71	14.1%	38	12.3%
Used antihypertensive medication in the past week^**^	279		172	
Yes	161	57.7%	97	56.4%
No	55	19.7%	34	19.8%
Unsure/unknown	59	21.1%	41	23.8%
Diabetes (DM)	696		460	
**Diagnosed with DM**	110	15.8%	69	15.0%
No prior diagnosis of DM	586	84.2%	391	85.0%
Regularly uses antidiabetic medication^*^	110		69	
Yes	80	72.7%	53	76.8%
No	20	18.2%	12	17.4%
Unsure/unknown	10	9.1%	4	5.8%
Used antidiabetic medication in the past week^**^	80		53	
Yes	58	72.5%	39	73.6%
No	8	10.0%	5	9.4%
Unsure/unknown	14	17.5%	9	17.0%
**Hemoglobinopaty**	696		457	
- Diagnosed with any hemoglobinopathy	1	0.1%	1	0.2%
**Human immunodeficiency virus (HIV)**	696		457	
- Diagnosed with HIV	5	0.7%	4	0.9%
**Family history of stroke (*n* = 696)**	687		457	
Yes	195	28.4%	113	24.7%
No	434	63.2%	306	67.0%
Unsure/unknown	58	8.4%	38	8.3%
**Tobacco smoking, current**	681		451	
Yes	34	5.0%	23	5.1%
No	647	95.0%	428	94.9%
**Alcohol use**	674		445	
Yes	37	5.5%	22	4.9%
No	637	94.5%	423	95.1%
**Physical exercise > 30 min daily**	688		457	
Yes	254	36.9%	181	39.6%
No	423	61.5%	266	58.2%
Unsure/unknown	11	1.6%	10	2.2%
**Diet with daily intake of**	687		432	
Fish	315	45.9%	226	52.3%
Meat	11	1.6%	9	2.1%
Leafy green vegetables	301	43.8%	209	48.4%
**Waist circumference (WC-mid)**				
Men	203		139	
Mean circumference (centimeter)	78.3	10.6	78.8	10.3
Increased (≥94 centimeter)	17	8.4%	13	9.4%
Women	224		131	
Mean circumference (centimeter)	84.1	14.9	84.3	15.1
Increased (≥80 centimeter)	138	61.6%	82	62.6%
**Stroke symptoms**	689		454	
Sudden onset	617	89.6%	409	90.1%
Limb weakness	449	65.2%	304	67.0%
Altered level of consciousness	304	44.1%	193	42.5%
Affected speech	426	61.8%	275	60.6%
Seizures	127	18.4%	76	16.7%

* Among those with a diagnosis.

** Among those who reported regularly being on medication.

### Stroke presentation and participant characteristics

Overall, 463 (64.3%) participants were experiencing a first-ever stroke, and 223 (31.0%) a recurrent stroke, while in 34 (4.7%) prior stroke status was unknown.

The median age of participants with any stroke was 62 years (53–70). Overall, 377 (52.2%) of strokes cases were women, 503 (72.3%) had a prior diagnosis of hypertension and 110 (15.8%) had a prior diagnosis of diabetes. Among those with a previous diagnosis of hypertension and who reported regularly receiving treatment, 161 (57.7%) had been using antihypertensive medication within the past 7 days leading up to stroke symptom onset. Hemoglobinopathies and HIV contributed very little to risk factor burden with 1 (0.1%) and 5 (0.7%) participants having been diagnosed, respectively.

Obesity assessed by WC-mid was present in 17 (8.4%) of men and 138 (61.6%) of women, and a total of 61.5% reported a physical activity level below 30 min/day. Median mNIHSS score was 19 (10–27) and 59.9% had a mNIHSS score above 15 (moderate to severe stroke).

### Stroke types

Of the patients with neuroimaging performed, evidence of acute/subacute ischemic stroke was present in 289 (62.8%), and 140 (30.4%) had evidence of ICH, while in 31 (6.7%) the underlying stroke pathology could not be determined (Table 4).

**Table 4:** In-hospital characteristics.

	**Any stroke (*****N*** = **720)**		**First-ever stroke (*****N*** = **463)**	
	***n*** **(or median)**	**% (or IQR)**	***n*** **(or median)**	**% (or IQR)**
**In-hospital status**				
**Stroke severity (mNIHSS score)**	670		447	
No symptoms (0)	13	1.9%	13	2.9%
Minor stroke (1–4)	62	9.3%	45	10.1%
Moderate stroke (5–15)	194	29.0%	148	33.1%
Moderate to severe stroke (16–20)	75	11.2%	50	11.2%
Severe stroke (>20)	326	48.7%	191	42.7%
Median mNIHSS score	19	10–27	17	9–27
**Neuroimaging**	720		463	
Non-contrast head CT or MRI^*^	460	63.9%	304	65.7%
**Stroke type**	460		304	
Acute/subacute ischemic infarct	289	62.8%	177	58.2%
Acute/subacute intracerebral hemorrhage	140	30.4%	97	31.9%
Chronic infarct	31	6.7%	30	9.9%

* Non-contrast computed tomography (n = 456) or magnetic resonance imaging (n = 4).

### First-ever stroke

Neuroimaging was undertaken in 304 (65.7%) of first-ever stroke patients with evidence of acute/subacute ischemic infarcts in 177 (58.2%), ICH in 97 (31.9%), and undetermined stroke type in 30 (9.9%).

Of all the first-ever strokes, 23 (5.1%) occurred in patients younger than 40 years; median age was 62 years (53–70), and 240 (51.8%) were women. Median mNIHSS score was 17 (9–27) and 241 (53.9%) had a mNIHSS score above 15.

## Discussion

These unique data show the stroke incidence is high in Zanzibar. Over 12 months 169.6 per 100.000 adult inhabitants sought hospital admission due to stroke, just below half of whom had symptoms of severe stroke. In one out of three who underwent neuroimaging an intracerebral hemorrhage was the underlying mechanism of the stroke, and among patients discharged from hospital alive two out of three had moderate-severe or severe disability.

Similar incidence studies in the SSA region have yielded a wide range of results, and study methodologies are not easily comparable due to difference in study population and inclusion criteria. However, health facility-based studies have found age-adjusted stroke incidence rates up to 260.1 per 100 000 adult population in Maputo, Mozambique (10) compared to which our study found even higher age-adjusted rate at 286.6 per 100.000 adult population. Younger women in our study were found to have higher age-standardized incidence rates than younger men for first-ever stroke. This is an observation that is not consistently found in previous studies, though it was also observed in the Maputo study (10). Hypertensive disorders of pregnancy appear to be very prevalent in Zanzibar (24, 25) and increase the risk of stroke later in life (26) which could contribute to the higher incidence rate among younger women. This poses a serious health concern and needs further investigation in future research. However, it also provides a window of opportunity to identify women at high risk and ensure adequate BP monitoring and control to reduce the risk of developing stroke later in life.

Hospital studies elsewhere in the region have found slightly higher ratios of ICH to ischemic strokes than in our study (10, 27–29), which might also reflect the more disabling and lethal nature of ICH and hence preference for hospitalization. At the same time, just below half of the participants in our study met the mNIHSS score criteria for severe stroke, meaning that people with mild or moderate symptoms of stroke also attended hospital for care.

The median age of first-ever stroke was 62 years, which is 10–15 years younger than in middle- and high-income countries (30, 31), confirming findings from other stroke studies in SSA (10, 31, 32) that stroke affects the working population with huge implications for the individual, their family, and the society (8).

The prevalence of diabetes at 15.8% was, not surprisingly, higher than in the Zanzibar background population at 3.9% (33), as there is a 2-fold increased risk of stroke for people with diabetes (1). However, when it comes to diabetes control, only roughly half of people with diabetes in our study had used diabetes medication within the week leading up to stroke onset. This is far fewer than expected (34, 35) and could be an indication of poorer medication adherence as an independent risk factor of stroke. A policy to screen, identify and treat patients with undiagnosed diabetes after stroke is required to reduce poor outcomes and risk of recurrent stroke in this patient group. At the same time, due attention must be paid to medication adherence across the spectrum of diseases contributing to stroke, including hypertension.

A prior study in Zanzibar showed a population prevalence of hypertension among adults of 29.8%, and great losses through the care cascade with three out of four persons with a hypertension diagnosis having used antihypertensive treatment within the past 2 weeks, but only one in three achieving adequate BP control (33). Our study showed roughly three out of four stroke patients had been diagnosed with hypertension, and less than one in three of those had used antihypertensive medication in the last week leading up to stroke onset. The reason for the low level of antihypertensive medication use compared to the background population could be due to an overrepresentation of people with complications of diabetes and hypertension, such as stroke, among those with poor medicine adherence. It is striking that even among those with recurrent stroke, use of antihypertensive medication to prevent a recurrent stroke was low, and further studies assessing barriers to engaging with stroke prevention services, including knowledge, and understanding of stroke and stroke prevention among patients, as well as care providers, are required.

The SIREN study from West Africa pointed to hypertension being the prime modifiable driver of the stroke burden in SSA (8, 36, 37), and this should be a strategic consideration for stroke prevention interventions. Individual- and population-based interventions for primordial, primary and secondary prevention of stroke, applying a life course perspective on behavioral as well as cardiometabolic risk factors, must be ambitious still pragmatic and both applicable and scalable in real life settings including the specific clinical reality of Zanzibar.

Hemoglobinopathies and HIV, as well as alcohol use, contributed minimally to the profile of known risk factors in our study, and stands in contrast to other studies from Tanzania (27, 28) and the continent (8). Central obesity was very common among the female study population. However, the mean corrected WC-mid for patients measured in supine position was several centimeters less than the mean WC-mid for patients in standing position (see Supplementary Table 3), which in turn was comparable to the mean WC-mid in the general population (35). It is hence likely that the true prevalence of central obesity among stroke patients is higher than our estimates, and future development of an optimal population-specific conversion factor, as well as cut-off, for WC-mid is needed.

## Strengths and limitations

This is the first study to investigate stroke incidence in Zanzibar. The study had a 12-month consecutive recruitment and a clearly defined denominator population with limited access to health facilities outside the study area for individuals presenting with acute stroke so this will have maximized our case ascertainment. According to routinely collected hospital data, the first wave of Covid-19 did not seem to impact the number of admitted stroke patients significantly as compared to the same months in previous years, and hospital capacity was not stretched due to covid-19 during the 12 months data collection was undertaken.

Nevertheless, the study also has limitations. Despite aiming to capture an entire population in a specific geographical location, our study is hospital-based, not community based. On the other hand, out of pocket payments are not required at the public hospitals in Zanzibar for available services including CT scan, which reduces one barrier to attending hospital services.

In the group of patients who did not undergo neuroimaging, the diagnosis of stroke was clinically based and hence a possibility of misclassification exists. Finally, we cannot rule out that non-participants of the study were more likely to be those with either minimal or severe stroke, which would introduce some bias in our findings on stroke severity and stroke types.

## Conclusion

In this prospective observational study of adults with stroke, we observed a high yearly age-standardized incidence of any stroke at 286.8/100.000 population. The high proportion of patients who prior to stroke onset had had contact with health services indicates a role for health facilities in risk factor management including improving medicine adherence for hypertension control.

## Data availability statement

The raw data supporting the conclusions of this article will be made available by the authors, upon reasonable request.

## Ethics statement

The study was approved by the Zanzibar Health Research Ethical Committee (ZAHREC/02/July/2019/47) and registered with ClinicalTrials.gov (NCT04095806). Written informed consent was obtained from all participants, or next of kin on their behalf.

## Author contributions

JJ, DC, KN, and RW co-conceived the study. JJ led the data collation, conducted the analysis, and wrote the first draft of the manuscript. RW is the guarantor of the work. All authors provided critical inputs on multiple iterations and have approved the final version.

## Conflict of interest

The authors declare that the research was conducted in the absence of any commercial or financial relationships that could be construed as a potential conflict of interest.

## Publisher's note

All claims expressed in this article are solely those of the authors and do not necessarily represent those of their affiliated organizations, or those of the publisher, the editors and the reviewers. Any product that may be evaluated in this article, or claim that may be made by its manufacturer, is not guaranteed or endorsed by the publisher.

## Supplementary material

Supplementary Material 1 Study proforma.

Supplementary Material 2 List of variables, definitions, sources of data, and assessment methods.

Supplementary Material 3 Corrected (supine) mid-waist circumference vs. standing mid-waist circumference.
