# Supplementary material for: Incidence and characteristics of stroke in Zanzibar–a hospital-based prospective study in a low-income island population
Source: Front Neurol. 2022 Jul 28;13:931915. doi: 10.3389/fneur.2022.931915 (PMC9366665; doi:10.3389/fneur.2022.931915)
Supplement: Supplementary Material 1 — Study proforma. [file Data_Sheet_1.PDF]

Area	Individual variabel	description	note

Before start of the interview, please fill in:

- ☐ Interviewer name ______________
- ☐ Date and time (first data entry) __ __ / __ __ / __ __
- ☐ Interviewee
  - ☐ patient
  - ☐ spouse or close relative *(living in same household)*
  - ☐ other:
- ☐ Patient Medical Record available for review
  - ☐ Yes
  - ☐ No
- Hospital ID (if any) ___________________________
- ☐ Patient unique ID *(ddmmyy,interviewerID, 0X)* __ __ __ __ __ __- __ __-__ __

Administrative and socio-economic&demographic data (patient interview)

- ☐ Names
  - First name ______________________________
  - Middle name ______________________________
  - Last name ______________________________
- ☐ Age
  - Date of birth (dd/mm/yyyy) __ __/__ __/__ __ __ __
  - Age (only if date of birth is unknown) __________
  - Approximate age (use milestones) __________
- ☐ Sex:
  - F
  - M
- ☐ Ph no: [list all phone numbers of patient] __________________________ ________________________
  - spouse or relatives phone number(s) __________________________ ________________________
- ☐ Address
  - landmark, street name or mtaa __________________________________
  - village __________________________________
  - shehia __________________________________
  - district ________________________________
- ☐ Occupation
  what describes your main occupation in the past year
  - ☐ unemployed
  - ☐ housewife
  - ☐ selfemployed (incl. farmer)
  - ☐ formally employed, non-govt
  - ☐ formally employed, government
  - ☐ student
  - ☐ retired / umri kubwa
  - ☐ unemployed, unable to work
  - ☐ unemployed, able to work
- ☐ Highest level of Education
  - ☐ no formal schooling
  - ☐ some primary schooling
  - ☐ completed primary school
  - ☐ completed secondary school (form 4)
  - ☐ completed higher education (form 6 or above)
- ☐ Civil status
  - ☐ never married
  - ☐ cohabiting/married (single wife)
  - ☐ cohabiting/married (polygameous marriage)
  - ☐ separated/divorced/widowed
  - ☐ other

☐ Living
where are you currently living

- ☐ Own house or apartment
- ☐ in rented house/apartment
- ☐ in relatives house/apartment/ family home
- ☐ in rented room
- ☐ occupying uninhabited structure/ watchman
- ☐ in friends house/room
- ☐ not known

☐ standards of living and assets

YES		NO
☐	electricity: connected to grid/ solar power	☐
☐	has own well or tap water on the compound	☐
☐	flush toilet, or improved latrine (slab)	☐
☐	tiled roof or corrugated iron sheets	☐
☐	flooring: tiles or concrete	☐
☐	electricity or gas used for cooking	☐
☐	owns motorcycle or car	☐
☐	owns a fridge	☐
☐	owns sheep, goat or cow /s	☐

☐ Do you have a medical insurance

- ☐ **Yes**
- ☐ **No**

Stroke onset and hospital admission (patient interview)

Neurological condition *before* stroke onset

modified Rankin scale (ask patient or relative - tick sc

Did you have any disability before the stroke onset

- ☐ 0 - no symptoms
- ☐ 1 - no significant disability despite symptoms
- ☐ 2 - slight disability
- ☐ 3 - moderate disability but able to walk without assis
- ☐ 4 - moderate disability, unable to walk without assist
- ☐ 5 - severe disability
- ☐ 9 - unknown status before admission

Symptoms

☐ neurological symptoms (subjectively)
which symptoms did you initially have?
[*let the patient first reply, then you probe*]

- ☐ altered level of consciousness
- ☐ vertigo, nausea
- ☐ weakness of limbs
- ☐ affected speech
- ☐ facial droop
- ☐ visual disturbances
- ☐ convulsion
- ☐ other:

☐ onset

- ☐ sudden
- ☐ gradual (over hours)
- ☐ unknown

Time of onset of first symptoms

A ☐ Stroke onset (date) __ __/__ __/__ __

- definite time of symptom onset ☐ __ __ : __ __ hrs (use 24 hrs)
- estimated onset time ☐ ☐ morning ☐noon ☐afternoon ☐evening ☐night
- unknown time of onset ☐

Initiel action taken

B ☐ First contact to the health system __ __/__ __/__ __ at __ __ : __ __ hrs.
(time of first arrival at any health facility after symptoms arised) *dd / mm / yyyy* *hh : mm*

- Where was the first contact made
  - ☐ Mnazi Mmoja Hosp (self referral) clinic
  - ☐ Mnazi Mmoja Hosp (self referral) E.R
  - ☐ Bububu Military Hosp
  - ☐ District (cottage) hospital
  - ☐ PHCU+/PHCU
  - ☐ private health facility
  - ☐ other:

- what did you do between **A** and **B** *(tick all relevant boxes)*
  - ☐ stayed indoor at home
  - ☐ received traditional treatment (eg herbal)
  - ☐ received massage by mkandaji (traditional masseur)
  - ☐ underwent spiritual healing
  - ☐ other, specify:____________________________

- admission time MMH ☐ __ __/__ __/__ __ at __ __ : __ __ hrs.
- admitted through
  - ☐ Emergency Room
  - ☐ Clinic / OPD
  - ☐ referred from other health facility
  - ☐ other route:_________________________

- admitted to (ward)
  - ☐ medical ward
  - ☐ ICU
  - ☐ fast track (private ward)
  - ☐ other ward

Previous medical history *[selfreported existing medical conditions]*

- **Previous stroke:**
  did you ever have a stroke before?
  - ☐ Yes
  - ☐ No
  - ☐ Unknown/unsure

  If Yes; nb of strokes ☐ ____
  date of last stroke __ __/__ __/__ __ __ __ or unknown ☐
  type of stroke (if known)
  - ☐ ischemic stroke
  - ☐ intracerebral hemorrhage
  - ☐ other/unknown

- **Previous transischemic attack (TIA):**
  did you have a TIA before?
  - ☐ Yes
  - ☐ No
  - ☐ Unsure/unknown

  if yes, number of TIA ☐ ___
  date of last TIA ☐ __ __/__ __/__ __ __ __ or unknown ☐

- **Did you prior to the stroke/admission suffer from any disease?**
  - ☐ No known past relevant medical history
  - ☐ Cognitive impairment/dementia
  - ☐ Diabetes (DM)
  - ☐ Dyslipidemia
  - ☐ HIV
  - ☐ Hypertension (HTN)
  - ☐ TB
  - ☐ Any heart condition: if yes tick the relevant box:
    - ❑ established (Ischemic) Heart Disease / previous AI
    - ❑ Heart Failure / CCF
    - ❑ Atrial fibrillation
    - ❑ RHD
  - ☐ any other relevant:____________________________

- ❑ Prosthetic valve
- ❑ any type of cardiomyopathy
- ❑ other heart condition

☐ **Did you regullarly use any prescribed Medication *before* current admission?**

❑ anticoagulant	Y/N/unknown
❑ antiplatelet	Y/N/unknown
❑ AntiHTN	Y/N/unknown
❑ Antilipid	Y/N/unknown
❑ oral hypoglycemic	Y/N/unknown
❑ insulin	Y/N/unknown
❑ antiTB	Y/N/unknown
❑ ARV	Y/N/unknown
❑ Others	Y/N/unknown
❑ NONE	*check patients notebook for details, or previous presc*

☐ **Did you use this medicine within the last 7 days before stroke onset?**

- ☐ Yes
- ☐ No
- ☐ Don't know/ cannot remember

Risk factors assessed (patient interview)

☐ **Family history of** stroke (parents, siblings, children)

- ☐ Yes
- ☐ No
- ☐ Don't know

☐ **current tobacco** smoking

- ☐ Yes
- ☐ No
- if yes ☐ *on average* how many cigarettes a day _________
- if yes ☐ have been smoking for how many years _______

☐ **exsmoker** (previously smoking tobacco)

- ☐ Yes
- ☐ No
- if yes ☐ how many years since quitting _________

☐ **Current alcohol use**
(within past year)

- ☐ Yes
- ☐ No
- if yes: drinking alcohol daily? ☐ Yes No ☐

☐ **Physical activity:** walking/ cycling/ swimming/ being physically active with increased pulse and respiration *on average* for at least 30 min a day every day

- ☐ Yes
- ☐ No
- ☐ Don't know

☐ sedentary for more than 8 hours a day
night time sleep excluded

- ☐ Yes
- ☐ No
- ☐ Don't know

☐ **diet :** on average, how often do you eat

**fish**	☐ daily	☐weekly	☐ monthly	☐I don't eat this at all	☐
**meat**	☐ daily	☐weekly	☐ monthly	☐I don't eat this at all	☐
**leafy green vegetables**	☐ daily	☐weekly	☐ monthly	☐I don't eat this at all	☐

☐ **psychological wellbeing**

have you been stressed, or recently suffered depression? (within the past month)

- ☐ Yes
- ☐ No
- ☐ Don't know

Admission to MMH (retrieved from the medical record)

- **tentative diagnosis at admission time**
  - ☐ stroke/CVA
  - ☐ Transcient Ischaemic Attack
  - ☐ hypertension (any form)
  - ☐ diabetes (any form)
  - ☐ admitted due to other condition, developed stroke ir
  - ☐ other admission diagnosis:

objective examination (retrieved from the medical record)

Vital signs etc

- Blood Pressure At arrival
  - ☐ Performed. BP ___ /___ mmHg
  - ☐ BP not measured
- Temperature at arrival
  - ☐ Performed. Temp = _____ C
  - ☐ Temp not measured
- Pulse at arrival (beats per minute):
  - ☐ Measured. Pulse rate ___ / min
  - ☐ Pulse rate not measured
- O2sat
  - ☐ O2 saturation ___ %
  - ☐ O2 saturation not measured
- BMI
  - ☐ BMI assessed ______ kg/m2
  - ☐ BMI not assessed
- Random blood sugar
  - ☐ Performed. RBG ______mmol/L
  - ☐ Not assessed

physical examination

- stet. C
  - ☐ Not described / not done
  - ☐ Regullar beat
  - ☐ Audible murmur
  - ☐ irregullar beat
  - ☐ N.A.
- neurological examination
  - ☐ full assessment ☐ partial assessment ☐ no assessr
- swallowing assessed
  - ☐ Yes ☐ No

Stroke Severity at Admission

- GCS (Glasgow Coma Score)
  - ☐ Yes: score ________
  - ☐ Not assessed
- NIHSS assessed at admission
  - ☐ Yes: score ________
  - ☐ Not assessed

Radiology (retrieved from the medical record)

Referral for imaging

- Date and time for referral
  - ☐ __ __/__ __/ __ __ __ __ at __ __: __ __
- Did the referral contain sufficient background information
  - ☐ Yes
  - ☐ No
  - ☐ Don't know
- Imaging performed
  - ☐ Yes
  - ☐ No: why not (list cause/s) _______________________
  - if yes ☐ date and time for first image __ __/__ __/__ __ __ _
- Type of imaging (tick all relevant)
  - ☐ CT
  - ☐ CTA/CTP

- [ ] MRI
- [ ] MRA/MRP
- [ ] other:

Workup (retrieved from the medical record)

X-ray

- [ ] CXR
  - ❑ not performed
  - ❑ performed : __ __/__ __/__ __ __ __ at __ __:__ .
  - ❑ normal
  - ❑ cardiomegaly
  - ❑ pulmonary edema
  - ❑ effusion
  - ❑ pneumonia
  - ❑ other (specify): ____________________________

- [ ] ECG
  - ❑ not performed
  - ❑ performed : __ __/__ __/__ __ __ __ at __ __:__ .
  - ❑ normal
  - ❑ new Infarct
  - ❑ old infarct
  - ❑ Atrial fibrillation / flutter
  - ❑ LVH
  - ❑ other: ________________________________

Ultrasound and echocardiography

- [ ] Not performed [skip following grey section]
- [ ] Performed [continue below]

- [ ] **Carotid ultrasound**
  - [ ] No
  - [ ] Yes

if yes, findings:

R int carotid

- ❑ Plaque
- ❑ normal findings only
- ❑ Calcified plaque
- ❑ aneurism
- ❑ ____ % stenosis
- ❑ other: specify _____________
- ❑ No abnormal findings

L int carotid

- ❑ Plaque
- ❑ normal findings only
- ❑ Calcified plaque
- ❑ aneurism
- ❑ ____ % stenosis
- ❑ other: specify _____________
- ❑ No abnormal findings

- [ ] **ECCO**
  - [ ] Not performed
  - [ ] Performed

if performed: findings

- [ ] left ventricular wall motion abnormalties
- [ ] LVEF
- [ ] vegetation
- [ ] Thrombus (describe location)
- [ ] valvular disease (describe valve)

☐ LVH
☐ R-L shunt
☐ other findings________________________________

Lab tests (from the medical record)

[tick box if ordered, and fill in results when these are available]

☐ ALT ________AST __________
☐ Aptt ( )____________
☐ triglycerides:________
☐ creatinine : _______________
☐ total cholesterol : _______HDL:_____LDL:_____
☐ electrolytes: ☐ Normal ☐ out of range
☐ FBG:_______
☐ Hematocrit ____________
☐ Hemoglobin ____________
☐ Platelet:______________
☐ PT/INR ( ) ____________
☐ Red cell count:__________
☐ S-albumin ____________
☐ TotalLeucocytCount________DifferentiatedLeucocytCount_______
☐ TSH _________
☐ urea ________
☐ urine analysis _________

☐ **other investigations on admission:**(describe results) ____________________________________

Treatment (from the medical record)

☐ **initial pharmaceutical treatment**

antiplatelet (e.g. aspirin)	☐	Yes
	☐	No
anticoagulant (e.g. warfarin)	☐	Yes
	☐	No
antiHTN	☐	Yes
	☐	No
antiglycemic drugs (tablest or insulin)	☐	Yes
	☐	No
statins	☐	Yes
	☐	No
prophylactic heparin im	☐	Yes
	☐	No
antibiotic	☐	Yes
	☐	No
other	☐	(specify)

☐ **supportive treatment**

- ❑ i.v. fluids
- ❑ NG tube
- ❑ Foleys Catheter
- ❑ mechanical ventilation
- ❑ ICU admission
- ❑ neurosurgery — describe ____________________________________
- ❑ other — specify what

RADIOLOGY REPORT (by research team radiologists)

Name on the image ________________________
Participant ID ________________________

Radiology department ______________________
Date of scan ______________________

Ischemic infarct:

- Hyperacute (0-6 hrs) ☐ yes
- Acute (6-24 hrs) ☐ yes
- Subacute (24 hrs - 14 days) ☐ yes

Intracerebral Hemorrhage

- Acute / subacute (0-14 days) ☐ yes

Chronic Infarct

- Chronic infarct(s) seen ☐ yes

Other diagnosis

- [free text] ________________________

Reported by Dr. ________________________

At discharge from hospital (from the medical record)

In-hospital complications / events

☐ None
☐ Following complications/ events occurred:

- ❑ Deep Venous Thrombosis
- ❑ (aspiration) pneumonia
- ❑ UTI
- ❑ GI hemorrhage
- ❑ Bedsore
- ❑ Re-Stroke
- ❑ unstabile angina pectoris / MI/CCF/pulmonary edema/cardiogenic shock
- ❑ phlebitis/cellulitis
- ❑ sepsis
- ❑ hematuria
- ❑ seizures
- ❑ Surgery
- ❑ Death
- ❑ Other [specify]
- ❑ foreign body aspiration

if death: record date of death, cause of death. End questionnaire here.

At day of discharge from hospital

☐ discharge date and time __ __/__ __ /__ __ __ __ at __ __:__ __

☐ Discharge diagnosis

- ☐ ischemic stroke
- ☐ hemorrhagic stroke
- ☐ TIA
- ☐ SAH
- ☐ other (specify)

☐ any new diagnosis given

- ☐ aphasia
- ☐ Atrial fibrillation / atrial flutter
- ☐ coronary artery disease / ischemic heart disease
- ☐ Diabetes Mellitus
- ☐ dyslipidemia
- ☐ hemi/paraplegia
- ☐ hypertension
- ☐ kidney failure
- ☐ other (specify)____________________

☐ BP at discharge / mmHg

Medications at discharge: *[tick box if medicine was prescribed/continued]*

- ❑ Antiplatelet
- ❑ Anti HTN

- ❑ Antilipid
- ❑ oral hypoglycemic
- ❑ antibiotic
- ❑ insulin
- ❑ supplements (vitB, …)
- ❑ Other : ______________________________

were all requested investigations performed?

- ☐ yes
- ☐ no
- ☐ unsure

was any planned assessment by physiotherapy done?

- ☐ yes
- ☐ no
- ☐ unsure

nursing care

was temperature monitored daily	☐ Yes<br>☐ No
was BP monitored daily	☐ *Yes*<br>☐ *No*
was intake-output / hydration status assessed	☐ Yes<br>☐ No
nutritional assessment was done	☐ Yes<br>☐ No

physiotherapy

- how many hours of physioterapy received while in hospital : ___________
- **speech therapy? ___________**

Any other important information captured (e.g. pregnancy status):

____
____
____

Condition at discharge (patient interview)

modified Rankin score (1-5)	☐ 0 - no symptoms<br>☐ 1 - no significant disability despite symptoms<br>☐ 2 - slight disability<br>☐ 3 - moderate disability, able to walk w/out assistance<br>☐ 4 - moderate disability, unable to walk w/out assistar<br>☐ 5 - severe disability<br>☐ 9 - unknown status
hemi / paraplegia	☐ no<br>☐ left side<br>☐ right side<br>☐ paraplegic
incontinence	☐ none<br>☐ bowel<br>☐ urine / catheterized<br>☐ unknown
if urine incontinence: discharged home with catheter?	☐ yes<br>☐ no

☐ dysphagia	☐	Yes
	☐	No
	☐	Unknown
☐ dysarthri/dysfasia/afasia *difficulties in communication (language)*	☐	Yes
	☐	No
	☐	Unknown
☐ other (describe)		

Discharged to:

- ❑ Home (pre-admission residence)
- ❑ other hospital
- ❑ relative's household
- ❑ Nursing home
- ❑ other (specify)

Care planning/next hospital appointment

☐ Is there a written plan for further treatment/care?	☐	Yes
	☐	unsure / don't know
	☐	No
☐ is antihypertensive treatment prescribed	☐	Yes
	☐	No
	☐	unsure/ don't know
☐ which clinic/hospital to attend, next appointment date		______________________________________
☐ is there a plan for rehabilitation	☐	unsure/ don't know
	☐	No
	☐	Yes
if yes	☐	physiotherapy appointment given on_________

Patient and family education, skills training

☐ received education about stroke	☐	Yes
	☐	No
	☐	unsure
☐ information on stroke prevention received	☐	Yes
	☐	No
	☐	unsure
☐ physiotherapy instructions received	☐	showed and instructed in exercises
	☐	oral instructions given
	☐	no instructions given
	☐	not known
	☐	not applicable for this patient
☐ instructions around medication given	☐	written instructions given
	☐	oral instructions given
	☐	no instructions given
	☐	not known
	☐	not applicable for this patient
☐ appointment for next clinic attendence given	☐	written instructions given
	☐	oral instructions given
	☐	no instructions given
	☐	not known
	☐	not applicable for this patient

Discharge situation		
☐ who requested discharge?	☐	patient
	☐	relative/caretaker
	☐	doctor
	☐	other (who)
☐ reason for requesting discharge if not d/c by doctor *(subjectively - list all answers)*	☐	improved condition, doesn’t have any problems
	☐	all investigations were done, no need to stay
	☐	do not believe hospital treatment can help
	☐	would like to try other treatment outside hospital
	☐	futile - no improvement, or in poor condition
	☐	no one to care for patient in hospital
	☐	other reason (which):___________________________
