# Supplementary material for: Incidence and characteristics of stroke in Zanzibar–a hospital-based prospective study in a low-income island population
Source: Front Neurol. 2022 Jul 28;13:931915. doi: 10.3389/fneur.2022.931915 (PMC9366665; doi:10.3389/fneur.2022.931915)
Supplement: Supplementary Material 2 — List of variables, definitions, sources of data, and assessment methods. [file Data_Sheet_2.PDF]

	Variable	Original values	Source of data	Assessment method	Definitions
On admission					
**Socio-demographic variables**					
	Age (years)	number / unknown	patient	interview	Age in whole years counted from date of birth when known; year of birth if date is unknown; or estimated using milestones such as age at the revolution, year of first marriage or childbirth. If age can not be estimated the value of the variable is ''unknown''
	Civil status	Single, never married/monogamous marriage / polygamous marriage/ divorced or widowed / unknown	patient	interview	Monogamous marriage: union between two people whether a religious ceremony /official documentation has taken place. Polygamous marriage: As above, but the male part has two or more wives / the female is sharing her husband with one or more co-wives.
	Educational attainment	unknown/no education / some primary education/completed primary education/completed secondary education/ completed higher education	patient	interview	Primary school completed: 6 or 7 years depending if schooling was in Tz or in Znz. Secondary school completed: Form three (old curriculum) or form four (new curriculum) completed

	Employment	government employee / non-goverment employee/self-employed/farmer/housewife/retired/unemployed, unable to work/unemployed, able to work	patient	interview	Housewife: when not engaged in any type of incomegenerating activities. Self-employed: any type of selfemployment including engaged in small scale incomegenerating activities such as making and selling bread, juice etc
	Residence		patient	interview	Main residence over the past 12 months. If more than one residence: Where was the majority of nights spent over the last 12 months
	Gender	female/male	patient	interview	Refers to biological gender
	Residence ownership	own residence/family home/ lives in relatives house/rents a house/rents a room / squatting, staff quarter /unknown	patient	interview	Own residence: Owns the house alone or with spouse. Family home: Owns the residence together with relatives e.g. siblings or uncles/aunties. Squatting: lives in an uninhabited house, typically uncompleted structure, without paying rent. Staff quarter: lives in accommodation provided by employer, does not have ownership of residence elsewhere
	Standards of living				
	Improved roofing	yes/no	patient	interview	Yes: tiles or corrugated iron sheets as roof (fully or partial). No: thatched grass roof
	Own water	yes/no	patient	interview	Yes: own well or connected to (public) water supply with water pipe/tap on the compound. No: Has to fetch water outside the compound

	Household electricity	yes/no	patient	interview	Yes: connected to grid, has generator or solar powered connection. No: not connected to electricity but might own a solarpowered torch etc.
	Floor of cement/tiles/	yes/no	patient	interview	Yes: Concret, cement, or tiles (fully or partial). No: compacted dirt floor, sand floor
	Type of stove for cooking	Clean / not clean	patient	interview	Clean: LP gas, electricity or solar powered stove. Not clean: any other type of energy used for cooking
	Type of toilet	Improved / not improved	patient	interview	Improved: water closet or improved pit latrine (concrete slab, covered pit). No: un-improved latrine, or open defecation
	Household assets				
	Fridge	yes/no	patient	interview	Yes: electric, gas or solar powered fridge. No: no functioning fridge
	Motorbike or car	yes/no	patient	interview	Yes: any type of motorbike, car or truck owned by a household member. No: No ownership of vehicle
	Livestock	yes/no	patient	interview	Yes: cattle, sheep, goat. No: poultry, donkey, dog
	Health Insurance	yes/no	patient	interview	Yes: any kind of health insurance paid privately, by employer, or through third party. No: Newly enrolled Zanzibar health insurance card (as there is no additional benefits to not having the insurance card)
**Health status prior to admission**					
Previous diagnoses					
	Diabetes	yes	patient	interview	Prior to stroke onset having been diagnosed by a health care professional to have Diabetes Mellitus

	Hemoglobinopathy	yes	patient	interview	Prior to stroke onset having been diagnosed by a health care professional to have sickle cell, thasallaemia, or any other hemoglobinopathy
	Hypertension	yes	patient	interview	Prior to stroke onset having been diagnosed by a health care professional to have hypertension
	Heart disease	yes	patient	interview	
	HIV	yes	patient	interview	
	Chronic kidney disease	yes	patient	interview	
	Tuberculosis	yes	patient	interview	
	Disability (mRankin)	0-5,9	patient	interview	0 : no disability 1: symptom no disability 2:minor disability 3: moderate disability, can walk without assistance 4: moderate disability, can walk with assistance 5: major disability, bedridden 9:unknown
	Previous stroke	yes/no/unknown	patient	interview	Yes: previous episode of stroke No: no known previous stroke episode. Unknown: unsure or unknown whether there has been a previous stroke episode
	Number of previous strokes	number	patient	interview	Number of stroke episodes excluding the current episode
	Date of last stroke episode	date	patient	interview	Full or partial date of last stroke episode, excluding the current episode
	Type of stroke	ischemic infarct / ICH / unknown	patient	interview	
	Previous TIA	yes/no/unknown	patient	interview	TIA: Stroke symptoms lasting less than 24 hours, typically few minutes / half an hour
	Number of TIAs	number	patient	interview	Number of TIA episodes
	Date of last TIA episode	date	patient	interview	Full or partial date of last TIA episode

Medication use					
	Attending clinic	yes/no/unknown	patient, clinic notes	interview, review of clinic notes	Regullar attending clinic services for the abovementioned pre-existing conditions
	Using prescription medication	yes/no/unknown	patient, clinic notes	interview, review of clinic notes	Regullarly using prescription medication for the above mentioned condition(s)
	Specific medications	number	patient, clinic notes	interview, review of clinic notes	1: Anticoagulant 2:Antiplatelet 3:HTN medication 4: lipid lowering medication 5:Oral antiglycemic medication 6: Insultin 7: TB medication 8: AntiRetroViral treatment 9: Other
	Medication use past week	number	patient	interview	1: within the last 7 days 2:NOT within the past 7 days 3:unsure/unknown
Other risk factors					
	Family history of stroke	yes/no/unknown	patient	interview	Family: birth mother, birth mother, or siblings from either of these parents
	Current smoking	yes/no/unknown	patient	interview	Yes: cigarette or smoking tobacco use within the past month
	number of cigarettes per day	number	patient	interview	Average number of cigarettes/ cigars/cigarillos/pipes of tobacco etc consumed per day
	years of smoking	number	patient	interview	estimated years since started smoking daily
	Previous smoking	yes/no/unknown	patient	interview	
	years since cessation	number	patient	interview	estimated number of years since smoke cessation
	Current alcohol use	yes/no/unknown	patient	interview	Yes: Alcohol use within the past month
	Daily alcohol intake	yes/no	patient	interview	daily: on average at least one unit of alcohol is consumed every day

	Physically active	yes/no/unknown	patient	interview	yes: on average daily 30 minutes or more of moderate physical activity like fast walking, cycling, exercise, swimming etc
	Sedentary	yes/no/unknown	patient	interview	yes: on average spending 8 hours or more sitting/lying down, for instance at an office desk or in bed, nighttime excluded
Diet					
	fish	daily/weekly/monthly /rarely/unknown	patient	interview	1:daily 2: weekly but not daily 3: monthly but not weekly 4: less than once a month 5: rarely/never
	meat	daily/weekly/monthly /rarely/unknown	patient	interview	1:daily 2: weekly but not daily 3: monthly but not weekly 4: less than once a month 5: rarely/never
	leafy green vegetables	daily/weekly/monthly /rarely/unknown	patient	interview	1:daily 2: weekly but not daily 3: monthly but not weekly 4: less than once a month 5: rarely/never
	Stress/depression	yes/no/unknown	patient	interview	Yes: Recently (within past two months) felt stress, depressed or anxious
**Stroke onset**					
	symptoms	change of consciousness/nausea or dizzyness/ loss of power or sensation / affected speech / seizures / facial droop / visual disturbance / other	patient	interview	
**Assessments**					
	Stroke severity (mNIHSS)	number	patient	Face-to-face assessment. Researcher certified in using the NIHSS instrument	0-4: minor stroke; 5-14: moderate stroke; 15-20: moderately severe stroke; 21-32: severe stroke

	Mid-waist circumference	centimetre	patient	mid-waist circumference (cm) measured by research assistant	WHO definition with abdominal circumference measured midway between the lowest rib and the iliac crest at the end of a normal expiration
	Hip circumference	centimeter	patient	Hip circumference (cm) measured by research assistant	As per WHO recommendation the measurement is taken around the widest portion of the buttocks
	Position during waist and hip measurement	supine/horisontal	patient	observation	Standing: standing on both feet with weight evently distributed. In case of disability an assistant or walking aid is supporting the patient. Supine: lying flat on the back on a bed or stretcher
**Neuroimaging**					
	Neuroimaging performed	yes/no/unsure	medical record, hospital register books, patient	review, interview	Yes: if neuroimaging was performed whether a copy of the image can be retrieved or not. No: no imaging performed
	Stroke type	hyperacute ischemic changes/ acute ischemic infarct / subacute ischemic infarct / acute or subacute ICH / chronic infarct/ nothing abnormal detected	Copy of non-contrast head CT or MRI	Reporting by research radiologist with neuroimaging experience. If any doubt a second review by neuroradiologist for final diagnosis	Stroke type and age at time of neuroimaging 1: hyperacute ischemic changes 0-6 hours. 2: acute ischemic infarct 6-24 hours. 3: subacute ischemic infarct 24 hours - 14 days. 4: acute or subacute intracerebral hemorrhage 0-14 days. 5:Chronic infarcts > 14 days, underlying pathology not determinable.
**Routine clinical, radiological, laboratory tests and treatments**					
Emergency room visit					
	Heart auscultation	yes/no	Patient medical record	Review of medical record	

	Neurological examination	full / partial/ none	Patient medical record	Review of medical record	
	Swallowing assessment	yes/no	Patient medical record	Review of medical record	
	Glasgow Coma Score	number / not assessed	Patient medical record	Review of medical record	
	mNIHSS on admission	number / not assessed	Patient medical record	Review of medical record	
	Was BP measured at arrival?	yes/no	Patient medical record	Review of medical record	
	SBP (mmHg)	number / not assessed	Patient medical record	Review of medical record	
	DBP (mmHg)	number / not assessed	Patient medical record	Review of medical record	
	Temperature at arrival (C)	number / not assessed	Patient medical record	Review of medical record	
	Pulse at arrival (beats per minute)	number / not assessed	Patient medical record	Review of medical record	
	Oxygen saturation at arrival (%)	number / not assessed	Patient medical record	Review of medical record	
	If not on room air: how many L of O2/min	number	Patient medical record	Review of medical record	
	Random blood glucose (mmol/L)	number / not assessed	Patient medical record	Review of medical record	
	CT/MRI requested	yes/no	Patient medical	Review of medical record	
	CT/MRI performed	yes/no	Patient medical	Review of medical record	
	Carotid ultrasound performed	yes/no	Patient medical record	Review of medical record	
	ECHO performed	yes/no	Patient medical	Review of medical record	

	ECG performed	yes/no	Patient medical record	Review of medical record	
Biochemestry					
	Platelet Count	number	Laboratory database	Review of laboratory database	$1000/mm^3$
	Prothrombin time	number	Laboratory database	Review of laboratory database	seconds
	Red cell count	number	Laboratory database	Review of laboratory database	$10^6/mm^3$
	Serum albumin	number	Laboratory database	Review of laboratory database	g/dL
	Total Leucocyte Count	number	Laboratory database	Review of laboratory database	$1000/mm^3$
	Thyroid stimulating hormone	number	Laboratory database	Review of laboratory database	micro-IU/mL
	Urea	number	Laboratory database	Review of laboratory database	mmol/L
	Urine analysis	number	Laboratory database	Review of laboratory database	standard reporting format
	Alanine aminotransferase (ALT)	number	Laboratory database	Review of laboratory database	U/L
	Aspartate aminotransferase (AST)	number	Laboratory database	Review of laboratory database	U/L
	Activated partial Thromboplastin time (APPT)	number	Laboratory database	Review of laboratory database	seconds
	Random blood sugar (on the ward)	number	Laboratory database	Review of laboratory database	mmol/L
	Creatinine	number	Laboratory database	Review of laboratory database	mmol/L
	Total cholesterol	number	Laboratory database	Review of laboratory database	mmol/L

	LDL cholesterol	number	Laboratory database	Review of laboratory database	mmol/L
	HDL cholesterol	number	Laboratory database	Review of laboratory database	mmol/L
	Electrolytes	number	Laboratory database	Review of laboratory database	mmol/L
	Fasting Blood Glucose	number	Laboratory database	Review of laboratory database	mmol/L
	Hematocrit	number	Laboratory database	Review of laboratory database	%
	Hemoglobin	number	Laboratory database	Review of laboratory database	g/dL
	Other tests	name and value	Laboratory database	Review of laboratory database	reported as per laboratory standards
Pharmaceutical treatment at hospital					
	Prescribed medication	Anticoagulant / antiplatelet / antihypertensive/ antidiabetic / statin / Heparin / antibiotics	Patient medical record	Review of medical record	Medication prescribed
	Supportive care	IV fluid/ Oxygen therapy / NGTube / mechanical ventilation / Foley's catheter / ICU admission	Patient medical record	Review of medical record	Supportive care prescribed during admission
	Other	text	Patient medical record	Review of medical record	
On discharge from hospital					

	Disability (mRankin)	0-5,9	patient	interview	0 : no disability 1: symptom no disability 2:minor disability 3: moderate disability, can walk without assistance 4: moderate disability, can walk with assistance 5: major disability, bedridden 9:unknown
	Paresis or paralysis		patient	interview	Any paresis/paralysis at time of discharge home
	Incontinence	no / fecal incontinence / incontinent for urine / unknown	patient	interview	Incontinent for urine covers both retention, overflow, and incontinence
	Discharged with Foley's catheter	yes/no	patient	interview	
	Discharged with functioning Naso-gastric tube	yes/no	patient	interview	
	Dysphagia	yes/no/unknown	patient	interview	
	Dysarthria	yes/no/unknown	patient	interview	
	Impaired ability to understand spoken language	yes/no/unknown	patient	interview	
Care planning					
	Discharge location	home / referred by medical doctor to other hospital / taken elsewhere by relatives for treatment / relatives household / nursing home / other	patient	interview	
	Informed about treatment/care plan post-discharge	yes/no/unsure	patient	interview	
	Prescription of anti-hypertensive medication	yes/no/unsure	patient	interview	
	Physioterapy appointment	Yes/no/unsure	patient	interview	
	Discharge on request by	Doctor / patient / relative / other	patient	interview	
Health education					

	Received education on stroke	yes/no/unsure	patient	interview	
	Informed about how to prevent stroke	yes/no/unsure	patient	interview	
	Education on exercises / physiotherapy	Demonstration and instructed in exercises / verbally instructed about exercises / no information / unknown / not relevant for this patient	patient	interview	
	Education on use of medication	Written instruction / verbally instruction / no information / unknown / not relevant for this patient	patient	interview	
	Given a date for follow up appointment at clinic	Yes, in writing / yes, verbally only / no / unknown / not relevant for this patient	patient	interview	
