# Supplementary material for: Incidence and characteristics of stroke in Zanzibar–a hospital-based prospective study in a low-income island population
Source: Front Neurol. 2022 Jul 28;13:931915. doi: 10.3389/fneur.2022.931915 (PMC9366665; doi:10.3389/fneur.2022.931915)
Supplement: Supplementary Material 3 — Corrected (supine) mid-waist circumference vs. standing mid-waist circumference. [file Data_Sheet_3.docx]

|  |  |  |  |  |  |  |  |  |
| --- | --- | --- | --- | --- | --- | --- | --- | --- |
| **Supplementary material 3**  Corrected mid-Waist Circumference (mid-WC) compared to standing measurement of mid-WC | | | | | | | | |
|  |  |  |  |  |  |  |  |  |
|  | (n) | Mean mid-WC | SD |  | Median mid-WC | IQR |  |  |
| **Women** |  |  |  |  |  |  |  |  |
| Supine (corrected mid-WC) | 198 | 84.31 | 14.87 |  | 84.32 | 72.65-90.43 |  |  |
| Standing | 26 | 87.21 | 22.82 |  | 84.00 | 72.00-95.00 |  |  |
| Total | 224 | 84.12 | 13.95 |  | 84.33 | 72.14-90.71 |  |  |
|  |  |  |  |  |  |  |  |  |
| **Men** |  |  |  |  |  |  |  |  |
| Supine (corrected mid-WC) | 168 | 77.58 | 10.38 |  | 76.27 | 71.19-83.89 |  |  |
| Standing | 35 | 81.95 | 11.28 |  | 80.00 | 76.00-87.00 |  |  |
| Total | 203 | 78.34 | 10.64 |  | 78.00 | 72.21-84.40 |  |  |
|  |  |  |  |  |  |  |  |  |
